# Association of Coexistent Hepatitis B Surface Antigen and Antibody With Severe Liver Fibrosis and Cirrhosis in Treatment-Naive Patients With Chronic Hepatitis B

**DOI:** 10.1001/jamanetworkopen.2022.16485

**Published:** 2022-06-13

**Authors:** Jian Wang, Weimao Ding, Jiacheng Liu, Yong Liu, Xiaomin Yan, Juan Xia, Weihua Wu, Bei Jia, Yuxin Chen, Dongmei Gao, Shuqin Hong, Xiaohong Wang, Li Wang, Xin Tong, Shengxia Yin, Zhaoping Zhang, Jie Li, Rui Huang, Chao Wu

**Affiliations:** 1 Department of Infectious Diseases, Nanjing Drum Tower Hospital, The Affiliated Hospital of Nanjing University Medical School, Nanjing, Jiangsu, China; 2 Institute of Viruses and Infectious Diseases, Nanjing University, Nanjing, Jiangsu, China; 3 Department of Hepatology, Huai’an No. 4 People’s Hospital, Huai’an, Jiangsu, China; 4 Department of Infectious Diseases, Nanjing Drum Tower Hospital Clinical College of Traditional Chinese and Western Medicine, Nanjing University of Chinese Medicine, Nanjing, Jiangsu, China; 5 Department of Laboratory Medicine, Nanjing Drum Tower Hospital, The Affiliated Hospital of Nanjing University Medical School, Nanjing, Jiangsu, China; 6 Community Work Office, Huai’an No. 4 People’s Hospital, Huai’an, Jiangsu, China; 7 Hospital Grade Creation Office, Huai’an No. 4 People’s Hospital, Huai’an, Jiangsu, China; 8 Department of Surgery, Huai’an No. 4 People’s Hospital, Huai’an, Jiangsu, China

## Abstract

**Importance:**

Coexistence of hepatitis B surface antigen (HBsAg) and antibody against HBsAg (anti-HBs) constitutes an atypical serological profile in chronic hepatitis B virus infection, and the association between coexistent HBsAg and anti-HBs with severe liver fibrosis and cirrhosis in patients with chronic hepatitis B (CHB) remains unclear.

**Objective:**

To investigate the association of coexistent HBsAg and anti-HBs with severe liver fibrosis and cirrhosis in patients with CHB.

**Design, Setting, and Participants:**

Consecutive treatment-naive patients with CHB from 2 medical institutions in China were enrolled between January 10, 2015, and March 31, 2021. Severe liver fibrosis and cirrhosis were identified using the aspartate transaminase (AST) to platelet ratio index (APRI), the fibrosis index based on 4 factors (FIB-4; factors comprise age, AST level, alanine aminotransferase [ALT] level, and platelet count), transient elastography, or ultrasonography. Data were analyzed from August 1, 2021, to April 15, 2022.

**Main Outcomes and Measures:**

The primary outcomes were rates of severe liver fibrosis and cirrhosis among patients with vs patients without coexistant HBsAg and anti-HBs. Severe liver fibrosis was defined as an APRI score of 1.5 or higher, a FIB-4 score of 3.25 or higher, or a liver stiffness measurement of 8 kPa or higher; cirrhosis was defined as an APRI score of 2.0 or higher, a FIB-4 score of 6.5 or higher, a liver stiffness measurement of 11 kPa or higher, or ultrasonographic findings suggestive of cirrhosis.

**Results:**

Of 6534 enrolled patients, 4033 patients (61.7%) were male, and the median (IQR) age was 41.0 (33.0-52.0) years. A total of 277 patients (4.2%) had coexistent HBsAg and anti-HBs. Patients with vs without anti-HBs were older (median [IQR], 46.0 [33.0-55.5] years vs 41.0 [33.0-52.0] years) and had a higher proportion of hepatitis B e antigen (HBeAg) positivity (123 of 277 patients [44.4%] vs 2115 of 6257 patients [33.8%]; *P* < .001), higher ALT levels (median [IQR], 45.1 [24.6-119.0] U/L vs 36.7 [22.0-77.0] U/L; *P* = .001) and AST levels (median [IQR], 35.0 [23.5-68.4] U/L vs 28.3 [21.6-51.0] U/L; *P* < .001), and lower platelet counts (median [IQR], 173.0 × 10^3^/μL [129.0-212.5 × 10^3^/μL] vs 185.0 × 10^3^/μL [141.0-224.0 × 10^3^/μL]; *P* = .004), albumin levels (median [IQR], 4.37 [4.11-4.56] g/dL vs 4.43 [4.17-4.61] g/dL; *P* = .02), and HBsAg levels (median [IQR], 2.8 log_10_ [1.6-3.4 log_10_] IU/mL vs 3.3 log_10_ [2.6-3.9 log_10_] IU/mL; *P* < .001). Compared with patients without anti-HBs, those with anti-HBs had higher APRI scores (median [IQR], 0.5 [0.3-1.4] vs 0.4 [0.3-0.9]; *P* < .001), FIB-4 scores (median [IQR], 1.4 [0.9-2.6] vs 1.1 [0.7-2.1]; *P* < .001), and liver stiffness values (median [IQR], 7.5 [6.2-9.8] kPa vs 6.3 [5.2-8.1] kPa; *P* = .003). Patients with anti-HBs also had higher proportions of severe liver fibrosis (102 of 277 patients [36.8%] vs 1397 of 6207 patients [22.5%]; *P* < .001) and cirrhosis (87 of 277 patients [31.4%] vs 1194 of 6213 patients [19.2%]; *P* < .001) compared with patients without anti-HBs. The coexistence of HBsAg and anti-HBs was independently associated with severe liver fibrosis (odds ratio [OR], 2.29; 95% CI, 1.56-3.38; *P* < .001) and cirrhosis (OR, 1.73; 95% CI, 1.12-2.68; *P* = .01) in the multivariate analysis. However, the association of coexistent HBsAg and anti-HBs with cirrhosis was only observed in patients with HBeAg negativity (OR, 1.66; 95% CI, 1.05-2.62; *P* = .03) and not in patients with HBeAg positivity (OR, 1.45; 95% CI, 0.87-2.43; *P* = .16).

**Conclusions and Relevance:**

In this cross-sectional study, the coexistence of HBsAg and anti-HBs was unusual in hepatitis B virus infection and was associated with more advanced liver diseases, such as severe liver fibrosis and cirrhosis, especially among patients with HBeAg negativity. These results suggest that close monitoring for liver fibrosis and cirrhosis is warranted in patients with CHB who have this serological profile.

## Key Points

**Question:**

Is the coexistence of hepatitis B surface antigen (HBsAg) and antibody against HBsAg (anti-HBs) associated with severe liver fibrosis and cirrhosis in patients with chronic hepatitis B (CHB)?

**Findings:**

In this cross-sectional study of 6534 patients with CHB, the coexistence of HBsAg and anti-HBs was independently associated with severe liver fibrosis and cirrhosis in patients with CHB, especially among those who had hepatitis B e antigen negativity.

**Meaning:**

This study’s findings suggest that coexistent HBsAg and anti-HBs is a rare serological pattern that may reflect a special status of infection and that close monitoring for liver fibrosis and cirrhosis is warranted in patients with CHB who have this serological profile.

## Teaser

This cross-sectional study assesses the association of coexistent hepatitis B surface antigen and antibody against hepatitis B surface antigen with severe liver fibrosis and cirrhosis in patients with chronic hepatitis B virus infection.

## Introduction

Chronic hepatitis B virus (HBV) infection is a major health burden, with approximately 290 million people estimated to be chronically infected worldwide.^1^ The natural history of chronic HBV infection is complex and variable.^2^ The serological markers of HBV are usually detected to identify different phases of HBV infection. The presence of hepatitis B surface antigen (HBsAg) is a typical sign of HBV infection and, in most patients with chronic hepatitis B (CHB), the antigen persists for a lifetime.^3^ Antibody against HBsAg (anti-HBs) is commonly regarded as neutralizing, and the appearance of anti-HBs suggests resolution of HBV infection.^4^ Previous studies have reported finding an uncommon serological pattern of coexisting HBsAg and anti-HBs in patients with CHB.^5,6,7,8^ The proportion of patients with anti-HBs is less than 10%.^5,7,8^ However, the potential mechanism and clinical importance of this serological pattern in patients with CHB remain unclear.

Coexistence of HBsAg and anti-HBs has been reported to be associated with the accumulation of HBsAg variants, especially in the *a* determinant within the major hydrophilic region.^7,9,10^ In patients with CHB, the coexistence of HBsAg and anti-HBs might increase the risk of hepatocellular cancer (HCC), which may be associated with disease progression and long-term prognosis.^6,11^ However, few studies have assessed the association of coexistent HBsAg and anti-HBs with severe liver fibrosis and cirrhosis. Therefore, we aimed to investigate the association of the coexistence of HBsAg and anti-HBs with severe liver fibrosis and cirrhosis in patients with CHB.

## Methods

### Patients

This retrospective cross-sectional study was conducted at 2 medical centers, Nanjing Drum Tower Hospital (Nanjing, China) and Huai’an No. 4 People’s Hospital (Huai’an, China), from January 10, 2015, to March 31, 2021. Data were analyzed from August 1, 2021, to April 15, 2022. A total of 6534 consecutive patients with CHB were enrolled from both centers. The study was approved by the ethics committees of Nanjing Drum Tower Hospital and Huai’an No. 4 People’s Hospital with a waiver of informed consent because the study was retrospective. This study adhered to the ethical guidelines of the Declaration of Helsinki^12^ and followed the Strengthening the Reporting of Observational Studies in Epidemiology (STROBE) reporting guideline for cross-sectional studies.

Chronic HBV infection was defined as HBsAg positivity for more than 6 months before enrollment. Exclusion criteria^1^ were nonalcoholic fatty liver disease (diagnosed by ultrasonography or transient elastography)^2^; other viral hepatitis infection, including hepatitis A (defined as immunoglobulin M positivity for antibody against hepatitis A virus), hepatitis C (defined as RNA positivity for hepatitis C virus), and hepatitis E (defined as immunoglobulin M positivity for antibody against hepatitis E virus)^3^; immune liver diseases (such as abnormal autoantibodies and chronic liver dysfunction, including antinuclear antibodies and mitochondrial antibodies)^4^; alcohol-related liver disease (with alcohol-related defined as alcohol consumption of ≥30 g/d for men or ≥20 g/d for women)^5^; hereditary and metabolic liver diseases (diagnosed by history of illness)^6^; HCC or other type of cancer before enrollment^7^; thrombocytopenia^8^; and receipt of antiviral treatment before enrollment.

### Data Acquisition and Assessment

Clinical data from the patient’s first visit to the hospital were collected. Serological markers were detected using commercial immunoassays (Abbott Laboratories). The dynamic range of HBsAg levels was 0.05 to 250.00 IU/mL. The samples were retested with a stepwise dilution of 1:20 to 1:1000 if the HBsAg level was greater than 250 IU/mL. The positivity of anti-HBs titers was defined as 10 mIU/mL or greater.

Severe liver fibrosis and cirrhosis were diagnosed using the aspartate aminotransferase (AST) to platelet ratio index (APRI), the fibrosis index based on 4 factors (FIB-4; factors comprise age, AST level, alanine aminotransferase [ALT] level, and platelet count), transient elastography, or abdominal ultrasonography. The APRI score was calculated as the patient’s AST level (measured in U/L [to convert to microkatals per liter, multiply by 0.0167]) divided by the upper limit of the normal AST level, then divided by the patient’s platelet count (measured in 10^3^/μL [to convert to ×10^9^/L, multiply by 1]); the quotient was then multiplied by 100.^11^ The FIB-4 score was calculated as the patient’s age (measured in years) multiplied by the patient’s AST level (measured in U/L [to convert to microkatals per liter, multiply by 0.0167]), then divided by the patient’s platelet count (measured in 10^3^/μL [to convert to ×10^9^/L, multiply by 1]); the quotient was then multiplied by the patient’s ALT level (measured in U/L [to convert to microkatals per liter, multiply by 0.0167]) raised to the 0.5 power.^13^ Patients who met any of the following criteria were diagnosed with severe liver fibrosis^1^: (1) APRI score of 1.5 or higher,^2^ (2) FIB-4 score of 3.25 or higher,^3^ or (3) liver stiffness measurement (LSM) of 8 kPa or higher.^14^ Patients who met any of the following criteria were diagnosed with cirrhosis^1^: (1) APRI score of 2.0 or higher,^2^ (2) FIB-4 score of 6.5 or higher,^3^ (3) LSM of 11 kPa or higher,^14^ or (4) ultrasonographic findings suggestive of cirrhosis.^4^ Measurement of LSM by transient elastography was performed using an image-guided detection system (FibroTouch; Wuxi Hisky Medical Technologies Co), which has been found to have accuracy in identifying liver fibrosis that is comparable with another commonly used detection system (FibroScan; Echosens).^15^

### Statistical Analysis

Continuous variables were reported as medians with IQRs, and categorical variables were reported as frequencies with percentages. Independent *t* tests were used to compare continuous variables with normal distribution, and Mann-Whitney *U* tests were used to compare continuous variables with skewed distribution data. Binary categorical variables were analyzed using χ^2^ tests, and ordinal categorical variables were analyzed using χ^2^ tests for trend. The risk factors for severe liver fibrosis were identified using binary logistic regression analysis. Odds ratios (ORs) with 95% CIs were calculated. Variables selected for the univariate analysis were sex, ALT, HBsAg, hepatitis B e antigen (HBeAg) status, coexistence of HBsAg and anti-HBs, HBV DNA, and the interaction between anti-HBs and HBeAg status. Differences were considered statistically significant at 2-tailed *P* < .05. Variables with significance of *P* < .05 in the univariate analysis were adjusted for in the multivariate analysis. The statistical analysis was conducted using IBM SPSS software, version 23.0 (IBM Corporation).

## Results

### Patient Characteristics

Of 16 526 patients with CHB, 9992 patients were excluded based on exclusion criteria and lack of sufficient data (Figure). The flowchart of patient selection for each cohort is shown in the eFigure in the Supplement. Of 6534 enrolled patients (median [IQR] age, 41.0 [33.0-52.0] years; 4033 [61.7%] male and 2501 [38.3%] female), 4948 patients were from Nanjing Drum Tower Hospital (Nanjing cohort), and 1586 patients were from Huai’an No. 4 People’s Hospital (Huai’an cohort) (eTable 1 in the Supplement). Compared with patients in the Nanjing cohort, those in the Huai’an cohort were older (median [IQR], 45.0 [34.0-54.0] years vs 40.0 [32.0-51.0] years) and more likely to be male (1042 of 1586 patients [65.7%] vs 2991 of 4948 patients [60.4%]), have HBeAg positivity (813 of 1586 patients [51.3%] vs 1425 of 4948 patients [28.8%]) and cirrhosis detected by ultrasonography (217 of 868 patients [25.0%] vs 338 of 3284 patients [10.3%]), have higher levels of ALT (median [IQR], 83.0 [41.0-222.0] U/L vs 30.8 [20.1-54.8] U/L), AST (median [IQR], 56.0 [32.0-127.0] U/L vs 25.7 [20.5-37.5] U/L), HBsAg (median [IQR], 3.4 log_10_ [2.9-3.9 log_10_] IU/mL vs 3.2 log_10_ [2.4-3.8 log_10_] IU/mL), and HBV DNA (median [IQR], 5.6 log_10_ [3.5-7.0 log_10_] IU/mL vs 2.9 log_10_ [2.7-5.5 log_10_] IU/mL), and have higher scores on the APRI (median [IQR], 1.1 [0.5-2.8] vs 0.3 [0.3-0.6]) and the FIB-4 (median [IQR], 2.0 [1.1-4.4] vs 1.0 [0.7-1.7]).

**Figure.**
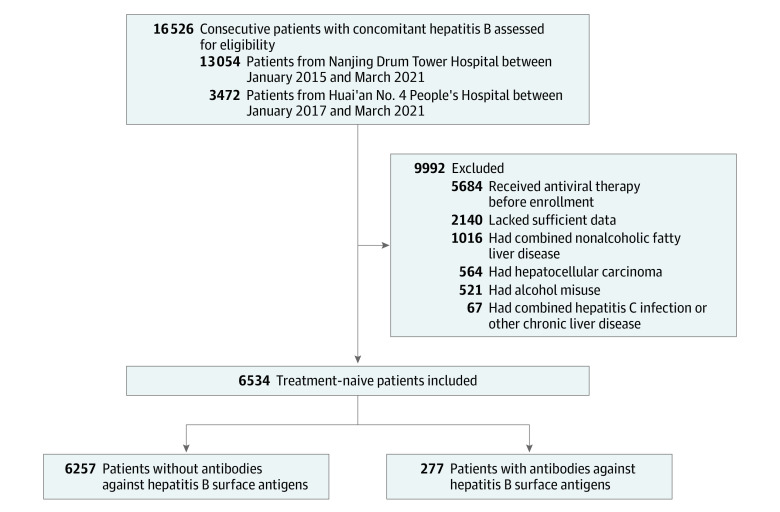
Flow Diagram of Participant Selection

Clinical characteristics were compared between enrolled and excluded patients (eTable 2 in the Supplement). In the Nanjing cohort, 184 of 4948 enrolled patients (3.7%) and 218 of 7367 excluded patients (3.0%) had anti-HBs. Compared with enrolled patients, excluded patients were older (median [IQR], 42.0 [33.0-53.0] years vs 40.0 [32.0-51.0] years) and more likely to be male (5660 of 8106 patients [69.8%] vs 2991 of 4948 patients [60.4%]) and have HBeAg positivity (2580 of 7284 patients [35.4%] vs 1425 of 4948 patients [28.8%]), cirrhosis detected by ultrasonography (437 of 2542 patients [17.2%] vs 338 of 3284 patients [10.3%]), higher levels of liver stiffness (median [IQR], 7.0 [5.8-9.1] kPa vs 6.4 [5.3-8.1] kPa) and AST (median [IQR], 26.5 [20.9-38.4] U/L vs 25.7 U/L [20.5-37.5] U/L), and higher scores on the APRI (median [IQR], 0.4 [0.3-0.7] vs 0.3 [0.3-0.6]) and FIB-4 (median [IQR], 1.2 [0.8-2.2] vs 1.0 [0.7-1.7]). However, enrolled patients had higher platelet counts (median [IQR], 189.5 × 10^3^/μL [152.0-226.0 × 10^3^/μL] vs 180.0 × 10^3^/μL [134.0-221.0 × 10^3^/μL]) and higher levels of HBV DNA (median [IQR], 2.9 log_10_ [2.7-5.5 log_10_] IU/mL vs 2.7 log_10_ [2.7-3.9 log_10_] IU/mL) than excluded patients.

In the Huai’an cohort, 93 of 1586 enrolled patients (5.9%) and 107 of 1673 excluded patients (6.4%) had anti-HBs (eTable 2 in the Supplement). Compared with enrolled patients, excluded patients were older (median [IQR], 52.0 [43.0-59.3] years vs 45.0 [34.0-54.0] years) and more likely to be male (1423 of 1886 patients [75.5%] vs 1042 of 1586 patients [65.7%]), have cirrhosis detected by ultrasonography (388 of 698 patients [55.6%] vs 217 of 868 patients [25.0%]), and have higher scores on the FIB-4 (median [IQR], 2.2 [1.2-4.9] vs 2.0 [1.1-4.4]). However, excluded patients were less likely to have HBeAg positivity (666 of 1673 patients [39.8%] vs 813 of 1586 patients [51.3%]) and had lower levels of ALT (median [IQR], 43.0 [26.0-104.0] U/L vs 83.0 [41.0-222.0] U/L), AST (median [IQR], 38.0 [25.0-78.0] U/L vs 56.0 [32.0-127.0] U/L), HBsAg (median [IQR], 3.1 log_10_ [2.3-3.6 log_10_] IU/mL vs 3.4 log_10_ [2.9-3.9 log_10_] IU/mL), and HBV DNA (median [IQR], 2.7 log_10_ [2.7-5.6 log_10_] IU/mL vs 5.6 log_10_ [3.5-7.0 log_10_] IU/mL) and lower scores on the APRI (median [IQR], 0.8 [0.4-1.9] vs 1.1 [0.5-2.8]) than enrolled patients.

Among all 6534 enrolled patients, 277 (4.2%) had anti-HBs (Table 1). The median (IQR) age was higher in patients with anti-HBs vs patients without anti-HBs (46.0 [33.0-55.5] years vs 41.0 [33.0-52.0] years; *P* = .005). Compared with patients without anti-HBs, those with anti-HBs had higher levels of ALT (median [IQR], 45.1 [24.6-119.0] U/L vs 36.7 [22.0-77.0] U/L; *P* = .001) and AST (median [IQR], 35.0 [23.5-68.4] U/L vs 28.3 [21.6-51.0] U/L; *P* < .001) and lower platelet counts (median [IQR], 173.0 × 10^3^/μL [129.0-212.5 × 10^3^/μL] vs 185.0 × 10^3^/μL [141.0-224.0 × 10^3^/μL]; *P* = .004) and albumin levels (median [IQR], 4.37 [4.11-4.56] g/dL vs 4.43 [4.17-4.61] g/dL; *P* = .02 [to convert to grams per liter, multiply by 10]). The proportion of HBeAg positivity among patients with anti-HBs was higher than that among patients without anti-HBs (123 of 277 patients [44.4%] vs 2115 of 6257 patients [33.8%]; *P* < .001). Median serum HBV DNA levels were also higher in patients with anti-HBs (median [IQR], 4.2 log_10_ [2.7-6.3 log_10_] IU/mL) vs patients without anti-HBs (median [IQR], 3.4 log_10_ [2.7-6.3 log_10_] IU/mL; *P* = .045). However, patients with anti-HBs had lower serum HBsAg levels (median [IQR], 2.8 log_10_ [1.6-3.4 log_10_] IU/mL) than patients without anti-HBs (median [IQR], 3.3 log_10_ [2.6-3.9 log_10_] IU/mL; *P* < .001).

**Table 1.** Comparison of Clinical Characteristics Among Patients With and Without Anti-HBs

Characteristic	Patients, No./total No. (%)		*P* value
	Without anti-HBs	With anti-HBs	
Total patients, No.	6257	277	NA
Age, median (IQR), y	41.0 (33.0-52.0)	46.0 (33.0-55.5)	.005
Sex			
Male	3874/6257 (61.9)	159/277 (57.4)	.13
Female	2383/6257 (38.1)	118/277 (42.6)	
Platelet count, median (IQR), × 10^3^/μL	185.0 (141.0-224.0)	173.0 (129.0-212.5)	.004
Missing, No.	34	0	NA
Total bilirubin, median (IQR), mg/dL	0.78 (0.58-1.09)	0.80 (0.61-1.15)	.08
Missing, No.	36	2	NA
ALT, median (IQR), U/L	36.7 (22.0-77.0)	45.1 (24.6-119.0)	.001
Missing, No.	17	0	NA
AST, median (IQR), U/L	28.3 (21.6-51.0)	35.0 (23.5-68.4)	<.001
Missing, No.	22	0	NA
GGT, median (IQR), U/L	25.7 (16.5-50.0)	30.0 (19.0-56.0)	.004
Missing, No.	69	4	NA
Albumin, median (IQR), g/dL	4.43 (4.17-4.61)	4.37 (4.11-4.56)	.02
Missing, No.	48	2	NA
HBsAg, log_10_ IU/mL			
Median (IQR)	3.3 (2.6-3.9)	2.8 (1.6-3.4)	<.001
Group			
<3	2233/6023 (37.1)	161/273 (59.0)	<.001
3 to <4	2516/6023 (41.8)	85/273 (31.1)	
≥4	1274/6023 (21.2)	27/273 (9.9)	
Missing, No.	234	4	NA
HBeAg status			
Positive	2115/6257 (33.8)	123/277 (44.4)	<.001
Negative	4142/6257 (66.2)	154/277 (55.6)	
HBV DNA, log_10_ IU/mL			
Median (IQR)	3.4 (2.7-6.3)	4.2 (2.7-6.3)	.045
Group			
<3	2468/5641 (43.8)	89/264 (33.7)	.07
3 to <5	1205/5641 (21.4)	66/264 (25.0)	
5 to <7	873/5641 (15.5)	66/264 (25.0)	
≥7	1095/5641 (19.4)	43/264 (16.3)	
Missing, No.	616	13	NA

Abbreviations: ALT, alanine aminotransferase; anti-HBs, antibody against hepatitis B surface antigen; AST, aspartate aminotransferase; GGT, γ-glutamyl transpeptidase; HBeAg, hepatitis B e antigen; HBsAg, hepatitis B surface antigen; HBV, hepatitis B virus; NA, not applicable.

SI conversion factor: To convert platelet count from ×10^3^/μL to ×10^9^/L, multiply by 1; to convert total bilirubin from milligrams per deciliter to micromoles per liter, multiply by 17.104; to convert ALT from units per liter to microkatals per liter, multiply by 0.0167; to convert AST from units per liter to microkatals per liter, multiply by 0.0167; to convert GGT from units per liter to microkatals per liter, multiply by 0.0167; to convert albumin from grams per deciliter to grams per liter, multiply by 10.

In addition, we compared the proportion of concurrent HCC between excluded patients with and without anti-HBs. A total of 564 patients with HCC were initially excluded, and data on HBsAg and anti-HBs status were not available for 35 patients (Figure). Among 529 patients with HCC who had data available on HBsAg and anti-HBs status, 490 patients (92.6%) did not have anti-HBs, and 39 patients (7.4%) had anti-HBs. A total of 39 of 316 patients (12.3%) with anti-HBs had HCC, which was a significantly higher proportion than that of patients without anti-HBs (490 of 6747 patients [7.3%]; *P* = .001).

### Severe Liver Fibrosis in Patients With vs Without Anti-HBs

Patients with vs without anti-HBs had higher median [IQR] APRI (0.5 [0.3-1.4] vs 0.4 [0.3-0.9]; *P* < .001) and FIB-4 (1.4 [0.9-2.6] vs 1.1 [0.7-2.1]; *P* < .001) scores (Table 2). According to APRI cutoff values, a greater proportion of patients with vs without anti-HBs had severe liver fibrosis (score ≥1.5; 65 of 277 patients [23.5%] vs 1040 of 6207 patients [16.8%]; *P* = .004) and cirrhosis (score ≥2.0; 50 of 277 patients [18.1%] vs 806 of 6207 patients [13.0%]; *P* = .02). According to FIB-4 cutoff values, a greater proportion of patients with vs without anti-HBs had severe liver fibrosis (score ≥3.25; 53 of 277 patients [19.1%] vs 910 of 6207 patients [14.7%]; *P* = .04), whereas the proportion of patients with cirrhosis was comparable (score ≥6.5; 20 of 277 patients [7.2%] vs 393 of 6207 patients [6.3%]; *P* = .55).

**Table 2.** Comparison of Liver Fibrosis Stages Between Patients With and Without Anti-HBs

Variable	Patients, No./total No. (%)		*P* value
	Without anti-HBs	With anti-HBs	
Total patients, No.	6257	277	NA
APRI score			
Median (IQR)	0.4 (0.3-0.9)	0.5 (0.3-1.4)	<.001
Severe liver fibrosis (≥1.5)	1040/6207 (16.8)	65/277 (23.5)	.004
Cirrhosis (≥2.0)	806/6207 (13.0)	50/277 (18.1)	.02
Missing, No.	50	0	NA
FIB-4 score			
Median (IQR)	1.1 (0.7-2.1)	1.4 (0.9-2.6)	<.001
Severe liver fibrosis (≥3.25)	910/6207 (14.7)	53/277 (19.1)	.04
Cirrhosis (≥6.5)	393/6207 (6.3)	20/277 (7.2)	.55
Missing, No.	50	0	NA
Cirrhosis detected on ultrasonography			
Yes	515/3991 (12.9)	40/161 (24.8)	<.001
Missing, No.	2266	116	NA
LSM, kPa			
Median (IQR)	6.3 (5.2-8.1)	7.5 (6.2-9.8)	.003
Severe liver fibrosis (≥8.0)	175/674 (26.0)	17/42 (40.5)	.04
Cirrhosis (≥11.0)	58/674 (8.6)	8/42 (19.0)	.02
Missing, No.	5583	235	NA

Abbreviations: anti-HBs, antibody against hepatitis B surface antigen; APRI, aspartate aminotransferase to platelet ratio index; FIB-4, fibrosis index based on 4 factors (age, aspartate transaminase level, alanine aminotransferase level, and platelet count); LSM, liver stiffness measurement; NA, not applicable.

Of 4152 patients with abdominal ultrasonographic data, 40 of 161 patients (24.8%) with anti-HBs had cirrhosis, which was significantly higher than the proportion among patients without anti-HBs (515 of 3991 patients [12.9%]; *P* < .001) (Table 2). Of 716 patients with available LSM data, patients with anti-HBs had higher LSM values (median [IQR], 7.5 [6.2-9.8] kPa) than those without anti-HBs (median [IQR], 6.3 [5.2-8.1] kPa; *P* = .003). According to LSM cutoff values, a greater proportion of patients with vs without anti-HBs had severe liver fibrosis (score ≥8.0; 17 of 42 patients [40.5%] vs 175 of 674 patients [26.0%]; *P* = .04) and cirrhosis (score ≥11.0; 8 of 42 patients [19.0%] vs 58 of 674 patients [8.6%]; *P* = .02). According to the definitions of severe fibrosis and cirrhosis, patients with anti-HBs had higher proportions of severe liver fibrosis (102 of 277 patients [36.8%] vs 1397 of 6207 patients [22.5%]; *P* < .001) and cirrhosis (87 of 277 patients [31.4%] vs 1194 of 6213 patients [19.2%]; *P* < .001) compared with patients without anti-HBs.

### HBV Features and Severity of Liver Fibrosis by HBeAg Status

Among 277 patients with anti-HBs, 123 (44.4%) were HBeAg positive, and 154 (55.6%) were HBeAg negative (Table 3). In the HBeAg-positive group, patients with anti-HBs had lower serum HBV DNA levels (median [IQR], 6.1 log_10_ [4.8-7.2 log_10_] IU/mL) than patients without anti-HBs (median [IQR], 7.1 log_10_ [5.5-7.8 log_10_] IU/mL; *P* < .001), whereas no significant difference in serum HBV DNA levels was observed between patients in the HBeAg-negative group with anti-HBs (median [IQR], 2.7 log_10_ [2.7-4.1 log_10_] IU/mL) vs without anti-HBs (median [IQR], 2.7 log_10_ [2.7-3.7 log_10_] IU/mL; *P* = .05). However, serum HBsAg levels remained lower in patients with vs without anti-HBs in both the HBeAg-positive group (median [IQR], 3.1 log_10_ [2.0-3.8 log_10_] IU/mL vs 4.0 log_10_ [3.4-4.6 log_10_] IU/mL; *P* < .001) and the HBeAg-negative group (median [IQR], 2.4 log_10_ [1.1-3.1 log_10_] IU/mL vs 3.0 log_10_ [2.1-3.5 log_10_] IU/mL; *P* < .001).

**Table 3.** Comparison of Clinical Features Between Patients With and Without Anti-HBs by HBeAg Status

Variable	Patients, No./total No. (%)					
	HBeAg positive			HBeAg-negative		
	Without anti-HBs (n = 2115)	With anti-HBs (n = 123)	*P* value	Without anti-HBs (n = 4142)	With anti-HBs (n = 154)	*P* value
**Virological parameters**						
HBsAg, log_10_ IU/mL						
Median (IQR)	4.0 (3.4-4.6)	3.1 (2.0-3.8)	<.001	3.0 (2.1-3.5)	2.4 (1.1-3.1)	<.001
Group						
<3	217/2043 (10.6)	52/120 (43.3)	<.001	2016/3980 (50.7)	109/153 (71.2)	<.001
3 to <4	826/2043 (40.4)	47/120 (39.2)		1690/3980 (42.5)	38/153 (24.8)	
≥4	1000/2043 (48.9)	21/120 (17.5)		274/3980 (6.9)	6/153 (3.9)	
Missing, No.	72	3	NA	162	1	NA
HBV DNA, log_10_ IU/mL						
Median (IQR)	7.1 (5.5-7.8)	6.1 (4.8-7.2)	<.001	2.7 (2.7-3.7)	2.7 (2.7-4.1)	.05
Group						
<3	186/1921 (9.7)	11/117 (9.4)	.002	2282/3720 (61.3)	78/147 (53.1)	.01
3 to <5	198/1921 (10.3)	22/117 (18.8)		1007/3720 (27.1)	44/147 (29.9)	
5 to <7	519/1921 (27.0)	47/117 (40.2)		354/3720 (9.5)	19/147 (12.9)	
≥7	1018/1921 (53.0)	37/117 (31.6)		77/3720 (2.1)	6/147 (4.1)	
Missing, No.	194	6	NA	422	7	NA
**Severe liver fibrosis and cirrhosis**						
APRI score						
Median (IQR)	0.7 (0.3-1.7)	0.9 (0.4-2.0)	.07	0.3 (0.2-0.6)	0.4 (0.3-0.9)	.008
Severe liver fibrosis (≥1.5)	586/2090 (28.0)	38/123 (30.9)	.49	454/4117 (11.0)	27/154 (17.5)	.01
Cirrhosis (≥2.0)	471/2090 (22.5)	31/123 (25.2)	.49	335/4117 (8.1)	19/154 (12.3)	.07
Missing, No.	25	0	NA	25	0	NA
FIB-4 score						
Median (IQR)	1.1 (0.7-2.3)	1.4 (0.8-2.6)	.03	1.1 (0.8-2.0)	1.4 (0.9-2.7)	.001
Severe liver fibrosis (≥3.25)	391/2090 (18.7)	26/123 (21.1)	.50	519/4117 (12.6)	27/154 (17.5)	.07
Cirrhosis (≥6.5)	179/2090 (8.6)	8/123 (6.5)	.43	214/4117 (5.2)	12/154 (7.8)	.16
Missing, No.	25	0	NA	25	0	NA
Cirrhosis detected by ultrasonography						
Yes	199/1292 (15.4)	21/73 (28.8)	.003	316/2699 (11.7)	19/88 (21.6)	.005
Missing, No.	823	50	NA	1443	66	NA
LSM, kPa						
Median (IQR)	6.3 (5.0-8.1)	6.9 (6.1-9.6)	.11	6.3 (5.3-8.0)	7.7 (6.3-10.5)	.003
Severe liver fibrosis (≥8.0)	51/192 (26.6)	6/18 (33.3)	.54	124/482 (25.7)	11/24 (45.8)	.03
Cirrhosis (≥11.0)	23/192 (12.0)	3/18 (16.7)	.56	35/482 (7.3)	5/24 (20.8)	.02
Missing, No.	1923	105	NA	3660	130	NA

Abbreviations: anti-HBs, antibody against hepatitis B surface antigen; APRI, aspartate aminotransferase to platelet ratio index; FIB-4, fibrosis index based on 4 factors (age, aspartate transaminase level, alanine aminotransferase level, and platelet count); HBeAg, hepatitis B e antigen; HBsAg, hepatitis B surface antigen; HBV, hepatitis B virus; LSM, liver stiffness measurement; NA, not applicable.

Of note, in the HBeAg-positive group, those with vs without anti-HBs had comparable APRI scores (median [IQR], 0.9 [0.4-2.0] vs 0.7 [0.3-1.7]; *P* = .07) and comparable proportions of severe liver fibrosis (score ≥1.5; 38 of 123 patients [30.9%] vs 586 of 2090 patients [28.0%]; *P* = .49) and cirrhosis (score ≥2.0; 31 of 123 patients [25.2%] vs 471 of 2090 patients [22.5%]; *P* = .49) (Table 3). However, in the HBeAg-negative group, patients with vs without anti-HBs had higher APRI scores (median [IQR], 0.4 [0.3-0.9] vs 0.3 [0.2-0.6]; *P* = .008) and a higher proportion of severe liver fibrosis (score ≥1.5; 27 of 154 patients [17.5%] vs 454 of 4117 patients [11.0%]; *P* = .01).

Although FIB-4 scores were higher in patients with anti-HBs vs patients without anti-HBs in both the HBeAg-positive group (median [IQR], 1.4 [0.8-2.6] vs 1.1 [0.7-2.3]; *P* = .03) and the HBeAg-negative group (median [IQR], 1.4 [0.9-2.7] vs 1.1 [0.8-2.0]; *P* = .001), the proportions of patients with vs without anti-HBs in the HBeAg-positive group who had severe liver fibrosis (score ≥3.25; 26 of 123 patients [21.1%] vs 391 of 2090 patients [18.7%]) and cirrhosis (score ≥6.5; 8 of 123 patients [6.5%] vs 179 of 2090 patients [8.6%]) were comparable with those of patients in the HBeAg-negative group (severe liver fibrosis: 27 of 154 patients [17.5%] vs 519 of 4117 patients [12.6%]; cirrhosis: 12 of 154 patients [7.8%] vs 214 of 4117 patients [5.2%]) (Table 3). According to abdominal ultrasonographic findings, the proportion of cirrhosis was also higher in patients with vs without anti-HBs in both the HBeAg-positive group (21 of 73 patients [28.8%] vs 199 of 1292 patients [15.4%]; *P* = .003) and the HBeAg-negative group (19 of 88 patients [21.6%] vs 316 of 2699 patients [11.7%]; *P* = .005).

The LSM values were comparable between the HBeAg-positive patients with vs without anti-HBs (median [IQR], 6.9 [6.1-9.6] kPa vs 6.3 [5.0-8.1] kPa; *P* = .11) (Table 3). In the HBeAg-positive group, no significant difference was observed in the proportion of patients with severe liver fibrosis (score ≥8.0; with vs without anti-HBs: 6 of 18 patients [33.3%] vs 51 of 192 patients [26.6%]; *P* = .54) or cirrhosis (score ≥11.0; with vs without anti-HBs: 3 of 18 patients [16.7%] vs 23 of 192 patients [12.0%]; *P* = .56). However, in the HBeAg-negative group, patients with vs without anti-HBs had significantly higher LSM values (median [IQR], 7.7 [6.3-10.5] kPa vs 6.3 [5.3-8.0] kPa; *P* = .003) and significantly higher proportions of severe liver fibrosis (11 of 24 patients [45.8%] vs 124 of 482 patients [25.7%]; *P* = .03) and cirrhosis (5 of 24 patients [20.8%] vs 35 of 482 patients [7.3%]; *P* = .02).

### Factors Associated With Severe Liver Fibrosis and Cirrhosis

In the univariate analysis, male sex (OR, 1.37; 95% CI, 1.22-1.55; *P* < .001), higher ALT levels (OR, 1.01; 95% CI, 1.01-1.01; *P* < .001), higher serum HBV DNA levels (OR, 1.32; 95% CI, 1.28-1.35; *P* < .001), coexistence of HBsAg and anti-HBs (OR, 2.01; 95% CI, 1.56-2.58; *P* < .001), HBeAg positivity (OR, 2.23; 95% CI, 1.99-2.51; *P* < .001), and the interaction between HBeAg and anti-HBs (OR, 2.57; 95% CI, 1.79-3.70; *P* < .001) were associated with severe liver fibrosis (Table 4). Coexistence of HBsAg and anti-HBs (OR, 2.29; 95% CI, 1.56-3.38; *P* < .001) remained the primary risk factor for severe liver fibrosis after adjusting for other indices in the multivariate analysis. Furthermore, the multivariate analysis revealed that the coexistence of HBsAg and anti-HBs was associated with cirrhosis (OR, 1.73; 95% CI, 1.12-2.68; *P* = .01).

**Table 4.** Analysis of Clinical Parameters Associated With Severe Liver Fibrosis and Cirrhosis

Variable	Severe liver fibrosis				Cirrhosis			
	Univariate analysis		Multivariate analysis		Univariate analysis		Multivariate analysis	
	OR (95% CI)	*P* value	OR (95% CI)	*P* value	OR (95% CI)	*P* value	OR (95% CI)	*P* value
Sex								
Female	1 [Reference]	<.001	1 [Reference]	.83	1 [Reference]	<.001	1 [Reference]	.58
Male	1.37 (1.22-1.55)		1.02 (0.87-1.19)		1.46 (1.28-1.66)		1.05 (0.89-1.23)	
Higher ALT level	1.01 (1.01-1.01)	<.001	1.01 (1.01-1.01)	<.001	1.01 (1.01-1.01)	<.001	1.01 (1.01-1.01)	<.001
Higher HBsAg level	1.00 (0.95-1.05)	.85	NA	NA	1.01 (0.96-1.07)	.62	NA	NA
Coexistence of HBsAg and anti-HBs								
No	1 [Reference]	<.001	1 [Reference]	<.001	1 [Reference]	<.001	1 [Reference]	.01
Yes	2.01 (1.56-2.58)		2.29 (1.56-3.38)		1.93 (1.48-2.50)		1.73 (1.12-2.68)	
HBeAg status								
Negative	1 [Reference]	<.001	1 [Reference]	.35	1 [Reference]	<.001	1 [Reference]	.37
Positive	2.23 (1.99-2.51)		0.91 (0.73-1.12)		2.34 (2.07-2.65)		1.11 (0.89-1.38)	
Interaction between HBeAg and anti-HBs	2.57 (1.79-3.70)	<.001	1.47 (0.79-2.71)	.22	3.06 (2.13-4.40)	<.001	1.18 (0.62-2.21)	.62
Higher HBV DNA level	1.32 (1.28-1.35)	<.001	1.05 (1.00-1.11)	.05	1.31 (1.27-1.35)	<.001	1.04 (0.99-1.09)	.17

Abbreviations: ALT, alanine aminotransferase; anti-HBs, antibody against hepatitis B surface antigen; HBeAg, hepatitis B e antigen; HBsAg, hepatitis B surface antigen; HBV, hepatitis B virus; NA, not applicable; OR, odds ratio.

A subgroup analysis of factors associated with severe liver fibrosis based on HBeAg status was performed. Coexistence of HBsAg and anti-HBs was also independently associated with severe liver fibrosis in both the HBeAg-positive group (OR, 1.89; 95% CI, 1.13-3.17; *P* = .02) and the HBeAg-negative group (OR, 2.28; 95% CI, 1.53-3.41; *P* < .001) (eTable 3 in the Supplement). A similar analysis revealed that the coexistence of HBsAg and anti-HBs was not independently associated with cirrhosis in the HBeAg-positive group (OR, 1.45; 95% CI, 0.87-2.43; *P* = .16), whereas coexistence was independently associated with cirrhosis in the HBeAg-negative group (OR, 1.66; 95% CI, 1.05-2.62; *P* = .03) (eTable 4 in the Supplement).

### Clinical Features and Severe Liver Fibrosis in Patients Who Received Liver Biopsy

A total of 303 patients had liver biopsy data available for analysis. Of those, 13 patients (4.3%) had anti-HBs (eTable 5 in the Supplement). Clinical features were comparable among patients with vs without anti-HBs (eg, HBsAg level: median [IQR], 3.3 log_10_ [3.0-3.8 log_10_] IU/mL vs 3.5 log_10_ [2.9-4.1 log_10_] IU/mL). A greater proportion of patients with vs without anti-HBs had severe liver fibrosis (8 of 13 patients [61.5%] vs 93 of 290 patients [32.1%]; *P* = .03) and cirrhosis (2 of 13 patients [15.4%] vs 9 of 290 patients [3.1%]; *P* = .02).

## Discussion

This cross-sectional study explored the epidemiological and clinical characteristics of patients with anti-HBs among a large real-world population with CHB. The prevalence of coexistent HBsAg and anti-HBs was 4.2%, which was comparable with the prevalence reported in previous studies.^5,7,8,16^

The virological features of the serological pattern revealed that a larger proportion of patients with vs without anti-HBs had HBeAg positivity, and patients with anti-HBs had higher HBV DNA levels than those without anti-HBs. Patients with HBeAg positivity typically have higher HBV DNA levels than those with HBeAg negativity.^17^ Therefore, to assess whether the higher HBV DNA levels observed may have been explained by the higher proportion of patients with anti-HBs who had HBeAg positivity, we performed a subgroup analysis by HBeAg status. We found that patients with anti-HBs had significantly lower HBV DNA levels, whereas there was no significant difference in HBV DNA levels in the HBeAg-negative group. Furthermore, the HBsAg levels in patients with anti-HBs were lower regardless of their HBeAg status. Anti-HBs is the major neutralizing antibody against HBV infection and may reflect immunity to HBV.^18^ Our findings suggest that serum anti-HBs may partly neutralize HBsAg and clear HBV in circulation. However, patients with anti-HBs had higher transaminase levels, suggesting that this serological pattern might be associated with active chronic hepatitis, despite anti-HBs being at a protective level.

The mechanism underlying the coexistence of HBsAg and anti-HBs in patients with CHB remains unclear. Lada et al^9^ found that coexistence of HBsAg and anti-HBs was associated with an increase in *a* determinant variability, leading to active chronic hepatitis; however, the underlying mechanism remained unclear. Several studies^19,20^ have reported that selective HBsAg immune escape variants may explain the uncommon serological pattern. First, variations or deletions in the pre-S/S gene and variations in the *a* determinant region and other regions of the S protein may be associated with the selection of HBsAg immune escape variants.^21,22,23^ The accumulation of variant middle and small surface proteins can activate the endoplasmic stress-signaling pathways in the endoplasmic reticulum of infected hepatocytes, thereby promoting oxidative DNA damage and genomic instability.^24^ Therefore, cytotoxic effects may also be an important reason that patients with anti-HBs have worse clinical outcomes.^24^ Second, Pu et al^5^ reported that genotype D was more common in patients with anti-HBs than without anti-HBs, and genotype D has a high level of genetic heterogeneity in the S gene.^25^ Third, our results revealed that patients with anti-HBs were older, which was consistent with findings from previous studies.^5,26,27^ Several previous studies reported that the incidence of amino acid variation increased with age.^28,29^ Moreover, the coexistence of HBsAg and anti-HBs may represent an immune recovery status of HBsAg seroconversion. The HBsAg and anti-HBs levels are relatively low when coexistent, and the status of coexistent HBsAg and anti-HBs may be transient.^30,31^ Therefore, the underlying mechanisms of coexistent HBsAg and anti-HBs appear to be multiple and complex and need to be validated in future research.

In addition, our results suggested that a greater proportion of patients with vs without anti-HBs had severe liver fibrosis and cirrhosis; thus, the presence of anti-HBs was identified as an independent risk factor for severe liver fibrosis and cirrhosis. In the subgroup analysis of patients with biopsy data, the proportions of severe liver fibrosis and cirrhosis were also higher among those with anti-HBs. Colson et al^26^ reported that patients with anti-HBs represented a higher proportion of those with severe liver fibrosis. However, only 13 patients with anti-HBs were included in that study.^26^ Previous studies reported that the coexistence of HBsAg and anti-HBs was a risk factor for HCC.^6,27,32^ Our study also found that the proportion of HCC was higher in patients with CHB and anti-HBs, which was consistent with findings from other studies.^6,27,32^ Moreover, we found that coexistent HBsAg and anti-HBs was associated with a higher proportion of liver cirrhosis, which may partially explain why the risk of HCC is higher in patients with anti-HBs, considering that the presence of liver cirrhosis is the single most important risk factor for HCC in patients with CHB.^33^ There may be several possible reasons for this result. First, patients with anti-HBs had high levels of HBV DNA and HBeAg positivity, which have always been regarded as risk factors for advanced disease among patients with CHB.^34^ Second, patients with anti-HBs had a longer duration of chronic HBV infection, as reflected in the median age of these patients, which was higher than that of patients without anti-HBs.^26^ However, patients with anti-HBs in the HBeAg-negative group had more severe liver fibrosis, whereas the severity of liver fibrosis was comparable between those with vs without anti-HBs in the HBeAg-positive group. The reason for this finding may be that patients with CHB who are HBeAg negative commonly have genome variations in the precore and/or basic core promoter regions.^35,36,37^ These variations may accelerate the host immune response to HBV-infected hepatocytes by increasing hepatocyte apoptosis and regeneration, leading to hepatocyte damage.^35,36,37^ However, the exact mechanism needs to be explored in the future.

### Limitations

This study has several limitations. First, owing to the lack of follow-up data, the duration of this unusual serological pattern and the association of coexistent HBsAg and anti-HBs with long-term outcomes among patients with CHB remain to be confirmed. Thus, our results require validation by further studies. Second, HBV genotypes were not available in our study, so the association of genotypes with coexistent HBsAg and anti-HBs warrants additional research. Furthermore, HBV genotypes in China are usually type B or C, and more studies are needed to validate our results among patients of other races and ethnicities.^38^ Third, the fibrosis stages were evaluated using composite measures of noninvasive tests. The extent of heterogeneity of different measures might have had implications for the study results. Thus, a sensitivity analysis of patients with available biopsy data was performed and revealed similar results. Fourth, owing to the lack of well-validated cutoff values for cirrhosis and for normal, moderate, and severe liver fibrosis on the APRI and FIB-4, we were not able to classify the severity of liver fibrosis as an ordinal variable with different levels and analyze the data using an ordinal logistic model.

## Conclusions

This cross-sectional study revealed that the coexistence of HBsAg and anti-HBs is a rare serological pattern that may reflect a special status of infection. Patients with anti-HBs, especially those with HBeAg negativity, may have a higher risk of severe liver fibrosis and cirrhosis. Therefore, close monitoring and attention to the potential development of liver fibrosis and cirrhosis during follow-up are warranted for patients with anti-HBs.

## Notes

Supplement. **eTable 1.** Comparison of Clinical Characteristics Between Enrolled Patients in 2 Cohorts **eTable 2.** Comparison of Clinical Characteristics Between Enrolled and Excluded Patients in Each Cohort **eTable 3.** Analysis of Clinical Parameters Associated With Severe Liver Fibrosis by HBeAg Status **eTable 4.** Analysis of Clinical Parameters Associated With Cirrhosis by HBeAg Status **eTable 5.** Comparison of Clinical Characteristics Between Patients With and Without Anti-HBs in Patients Who Underwent Liver Biopsy **eFigure.** Flowchart of Patient Selection in Each Cohort
