# Supplementary material for: Association of Coexistent Hepatitis B Surface Antigen and Antibody With Severe Liver Fibrosis and Cirrhosis in Treatment-Naive Patients With Chronic Hepatitis B
Source: JAMA Netw Open. 2022 Jun 13;5(6):e2216485. doi: 10.1001/jamanetworkopen.2022.16485 (PMC9194671; doi:10.1001/jamanetworkopen.2022.16485)
Supplement: Supplement. — eTable 1. Comparison of Clinical Characteristics Between Enrolled Patients in 2 Cohorts eTable 2. Comparison of Clinical Characteristics Between Enrolled and Excluded Patients in Each Cohort eTable 3. Analysis of Clinical Parameters Associated With Severe Liver Fibrosis by HBeAg Status eTable 4. Analysis of Clinical Parameters Associated With Cirrhosis by HBeAg Status eTable 5. Comparison of Clinical Characteristics Between Patients With and Without Anti-HBs in Patients Who Underwent Liver Biopsy eFigure. Flowchart of Patient Selection in Each Cohort [file jamanetwopen-e2216485-s001.pdf]

Supplementary Online Content

Wang J, Ding W, Liu J, et al. Association of coexistent hepatitis B surface antigen and antibody with severe liver fibrosis and cirrhosis in treatment-naive patients with chronic hepatitis B. *JAMA Netw Open*. 2022;5(6):e2216485. doi:10.1001/jamanetworkopen.2022.16485

eTable 1. Comparison of Clinical Characteristics Between Enrolled Patients in 2 Cohorts

eTable 2. Comparison of Clinical Characteristics Between Enrolled and Excluded Patients in Each Cohort

eTable 3. Analysis of Clinical Parameters Associated With Severe Liver Fibrosis by HBeAg Status

eTable 4. Analysis of Clinical Parameters Associated With Cirrhosis by HBeAg Status

eTable 5. Comparison of Clinical Characteristics Between Patients With and Without Anti-HBs in Patients Who Underwent Liver Biopsy

eFigure. Flowchart of Patient Selection in Each Cohort

This supplementary material has been provided by the authors to give readers additional information about their work.

© 2022 Wang J et al. *JAMA Network Open*.

eTable 1. Comparison of Clinical Characteristics Between Enrolled Patients in 2 Cohorts

Variables	Nanjing cohort (n=4,948)	Huai'an cohort (n=1,586)	P value
**Age (yr)**	40.0 (32.0, 51.0)	45.0 (34.0, 54.0)	<.001
**Male (%)**	2,991 (60.4)	1,042 (65.7)	<.001
**PLT (×10^3/μL)**	189.5 (152.0, 226.0)	157.0 (104.3, 205.0)	<.001
**Not available**	0	34	
**ALT (U/L)**	30.8 (20.1, 54.8)	83.0 (41.0, 222.0)	<.001
**Not available**	0	17	
**AST (U/L)**	25.7 (20.5, 37.5)	56.0 (32.0, 127.0)	<.001
**Not available**	0	22	
**HBsAg (Log_{10} IU/ml)**	3.2 (2.4, 3.8)	3.4 (2.9, 3.9)	<.001
**Group**			<.001
**<3**	1,936 (41.1)	458 (28.9)	
**3 to <4**	1,798 (38.2)	803 (50.6)	
**≥4**	976 (20.7)	325 (20.5)	
**Not available**	238	0	
**Anti-HBs positive (%)**	184 (3.7)	93 (5.9)	<.001
**Not available**	0	0	
**HBeAg positive (%)**	1,425 (28.8)	813 (51.3)	<.001
**Not available**	0	0	
**HBV DNA (Log_{10} IU/ml)**	2.9 (2.7, 5.5)	5.6 (3.5, 7.0)	<.001
**Group**			<.001

© 2022 Wang J et al. *JAMA Network Open*.

**<3**	2,265 (51.0)	292 (20.0)	
**3 to <5**	959 (21.6)	312 (21.3)	
**5 to <7**	457 (10.3)	482 (33.0)	
**≥7**	762 (17.2)	376 (25.7)	
**Not available**	505	124	
**APRI**	0.3 (0.3, 0.6)	1.1 (0.5, 2.8)	<.001
**APRI ≥1.5 (%)**	484 (9.8)	621 (40.4)	<.001
**APRI ≥2.0 (%)**	351 (7.1)	505 (32.9)	<.001
**Not available**	0	50	
**FIB-4**	1.0 (0.7, 1.7)	2.0 (1.1, 4.4)	<.001
**FIB-4 ≥3.25 (%)**	453 (9.2)	510 (33.2)	<.001
**FIB-4 ≥6.5 (%)**	173 (3.5)	240 (15.6)	<.001
**Not available**	0	50	
**Cirrhosis by ultrasound**	338 (10.3)	217 (25.0)	<.001
**Not available**	1664	718	
**Liver stiffness (kPa)**	6.4 (5.3, 8.1)	-	
**Liver stiffness ≥8.0 (%)**	192 (26.8)	-	
**Liver stiffness ≥11.0 (%)**	66 (9.2)	-	
**Not available**	4,232	1,586	

ALT, alanine aminotransferase; anti-HBs, hepatitis B core antibody; APRI, aspartate aminotransferase to platelet ratio index; AST, aspartate aminotransferase; FIB-4, fibrosis index based on the four factors; HBeAg, hepatitis B e antibody; HBsAg, hepatitis B surface antigen; HBV, Hepatitis B virus; PLT, platelet.

© 2022 Wang J et al. *JAMA Network Open*.

eTable 2. Comparison of Clinical Characteristics Between Enrolled and Excluded Patients in Each Cohort

Variables	Nanjing cohort				Huai'an cohort		
	Enrolled patients (n=4,948)	Excluded patients (n=8,106)	P value		Enrolled patients (n=1,586)	Excluded patients (n=1,886)	P value
**Age (yr)**	40.0 (32.0, 51.0)	42.0 (33.0, 53.0)	<.001		45.0 (34.0, 54.0)	52.0 (43.0, 59.3)	<.001
**Male (%)**	2,991 (60.4)	5,660 (69.8)	<.001		1,042 (65.7)	1,423 (75.5)	<.001
**PLT (×10^3/μL)**	189.5 (152.0, 226.0)	180.0 (134.0, 221.0)	<.001		157.0 (104.3, 205.0)	149.0 (95.0, 207.3)	.16
**Not available**	0	2,816			34	152	
**ALT (U/L)**	30.8 (20.1, 54.8)	31.1 (20.3, 54.4)	.85		83.0 (41.0, 222.0)	43.0 (26.0, 104.0)	<.001
**Not available**	0	669			17	94	
**AST (U/L)**	25.7 (20.5, 37.5)	26.5 (20.9, 38.4)	.005		56.0 (32.0, 127.0)	38.0 (25.0, 78.0)	<.001
**Not available**	0	708			22	97	
**HBsAg (Log_{10} IU/ml)**	3.2 (2.4, 3.8)	3.2 (2.4, 3.7)	.002		3.4 (2.9, 3.9)	3.1 (2.3, 3.6)	<.001
**Group**			<.001				<.001
**<3**	1,936 (41.1)	2,910 (41.9)			458 (28.9)	725 (44.6)	
**3 to <4**	1,798 (38.2)	2,916 (42.0)			803 (50.6)	710 (43.7)	
**≥4**	976 (20.7)	1,117 (16.1)			325 (20.5)	190 (11.7)	
**Not available**	238	1,163			0	261	
**anti-HBs positive (%)**	184 (3.7)	218 (3.0)	.02		93 (5.9)	107 (6.4)	.53
**Not available**	0	739			0	213	
**HBeAg positive (%)**	1,425 (28.8)	2,580 (35.4)	<.001		813 (51.3)	666 (39.8)	<.001
**Not available**	0	822			0	213	

© 2022 Wang J et al. *JAMA Network Open*.

**HBV DNA (Log$_{10}$ IU/ml)**	2.9 (2.7, 5.5)	2.7 (2.7, 3.9)	<.001		5.6 (3.5, 7.0)	2.7 (2.7, 5.6)	<.001
**Group**			<.001				<.001
**<3**	2,265 (51.0)	4,552 (64.2)			292 (20.0)	817 (53.9)	
**3 to <5**	959 (21.6)	1,219 (17.2)			312 (21.3)	251 (16.6)	
**5 to <7**	457 (10.3)	615 (8.7)			482 (33.0)	276 (18.2)	
**≥7**	762 (17.2)	709 (10.0)			376 (25.7)	171 (11.3)	
**Not available**	505	1,011			124	371	
**APRI**	0.3 (0.3, 0.6)	0.4 (0.3, 0.7)	<.001		1.1 (0.5, 2.8)	0.8 (0.4, 1.9)	<.001
**APRI ≥1.5 (%)**	484 (9.8)	569 (11.1)	.03		621 (40.4)	531 (31.8)	<.001
**APRI ≥2.0 (%)**	351 (7.1)	386 (7.5)	.38		505 (32.9)	408 (24.4)	<.001
**Not available**	0	2,993			50	214	
**FIB-4**	1.0 (0.7, 1.7)	1.2 (0.8, 2.2)	<.001		2.0 (1.1, 4.4)	2.2 (1.2, 4.9)	.006
**FIB-4 ≥3.25 (%)**	453 (9.2)	764 (14.9)	<.001		510 (33.2)	593 (35.5)	.18
**FIB-4 ≥6.5 (%)**	173 (3.5)	291 (5.7)	<.001		240 (15.6)	293 (17.5)	.15
**Not available**	0	2,993			50	214	
**Cirrhosis by ultrasound**	338 (10.3)	437 (17.2)	<.001		217 (25.0)	388 (55.6)	<.001
**Not available**	1,664	5,564			718	1,188	
**Liver stiffness (kPa)**	6.4 (5.3, 8.1)	7.0 (5.8, 9.1)	<.001		-	-	
**Liver stiffness ≥8.0 (%)**	192 (26.8)	214 (35.8)	<.001		-	-	
**Liver stiffness ≥11.0 (%)**	66 (9.2)	90 (15.1)	.001		-	-	
**Not available**	4,232	7,508			1,586	1,886	

ALT, alanine aminotransferase; anti-HBs, hepatitis B core antibody; APRI, aspartate aminotransferase to platelet ratio index; AST, aspartate aminotransferase; FIB-4, fibrosis index based on the four factors; HBeAg, hepatitis B e antibody; HBsAg, hepatitis B surface antigen; HBV, Hepatitis B virus; PLT, platelet.

© 2022 Wang J et al. *JAMA Network Open*.

eTable 3. Analysis of Clinical Parameters Associated With Severe Liver Fibrosis by HBeAg Status

Variables	HBeAg positive				HBeAg negative			
	Univariate analysis		Multivariate analysis		Univariate analysis		Multivariate analysis	
	OR (95% CI)	P value	OR (95% CI)	P value	OR (95% CI)	P value	OR (95% CI)	P value
**Sex**								
**Female**	Reference				Reference			
**Male**	1.321 (1.096, 1.592)	0.004	1.397 (1.096,1.779)	.007	1.389 (1.178, 1.638)	<0.001	1.165 (0.950, 1.428)	.14
**ALT (U/L)**	1.010 (1.009, 1.011)	<0.001	1.010 (1.009, 1.012)	<.001	1.014 (1.012, 1.015)	<0.001	1.010 (1.008, 1.012)	<.001
**HBsAg (Log_{10} IU/ml)**	0.468 (0.416, 0.527)	<0.001	0.381 (0.327, 0.445)	<.001	0.989 (0.923, 1.058)	0.743		
**HBsAg/anti-HBs coexistence**								
**No**	Reference				Reference			
**Yes**	1.584 (1.096, 2.289)	0.014	1.891 (1.126, 3.174)	.02	2.198 (1.551, 3.114)	<0.001	2.279 (1.525, 3.407)	<.001
**HBV DNA (Log_{10} IU/ml)**	0.997 (0.948, 1.049)	0.921			1.827 (1.720, 1.942)	<0.001	1.387 (1.289, 1.492)	<.001

ALT, alanine aminotransferase; HBeAg, hepatitis B e antibody; HBsAg, hepatitis B surface antigen; HBV, Hepatitis B virus; OR, odds ratio; PLT, platelet.

© 2022 Wang J et al. *JAMA Network Open*.

eTable 4. Analysis of Clinical Parameters Associated With Cirrhosis by HBeAg Status

Variables	HBeAg positive				HBeAg negative			
	Univariate analysis		Multivariate analysis		Univariate analysis		Multivariate analysis	
	OR (95% CI)	P value	OR (95% CI)	P value	OR (95% CI)	P value	OR (95% CI)	P value
**Sex**								
**Female**	Reference				Reference			
**Male**	1.541 (1.266, 1.877)	<0.001	1.095 (0.845, 1.418)	.49	1.365 (1.143, 1.631)	0.001	1.110 (0.889, 1.386)	.36
**ALT (U/L)**	1.007 (1.007, 1.008)	<0.001	1.008 (1.007, 1.009)	<.001	1.011 (1.010, 1.013)	<0.001	1.008 (1.007, 1.009)	<.001
**HBsAg (Log_{10} IU/ml)**	0.448 (0.397, 0.506)	<0.001	0.393 (0.328, 0.471)	<.001	1.043 (0.967, 1.125)	0.272		
**HBsAg/anti-HBs coexistence**								
**No**	Reference				Reference			
**Yes**	1.857 (1.282, 2.689)	0.001	1.450 (0.867, 2.425)	.16	1.719 (1.168, 2.530)	0.006	1.656 (1.048, 2.617)	.03
**HBV DNA (Log_{10} IU/ml)**	0.940 (0.893, 0.990)	0.018	0.961 (0.889, 1.038)	.31	1.902 (1.784, 2.027)	<0.001	1.469 (1.363, 1.585)	<.001

ALT, alanine aminotransferase; HBeAg, hepatitis B e antibody; HBsAg, hepatitis B surface antigen; HBV, Hepatitis B virus; OR, odds ratio; PLT, platelet.

© 2022 Wang J et al. *JAMA Network Open*.

eTable 5. Comparison of Clinical Characteristics Between Patients With and Without Anti-HBs in Patients Who Underwent Liver Biopsy

Variables	Patients without anti-HBs (n=290)	Patients with anti-HBs (n=13)	P value
**Age (yr)**	42.0 (35.0, 51.0)	41.0 (31.5, 53.0)	.77
**Male (%)**	183 (63.1)	10 (76.9)	.31
**PLT (×10^3/μL)**	179.0 (146.0, 218.5)	180.0 (122.0, 217.0)	.66
**ALT (U/L)**	37.0 (23.7, 55.0)	40.0 (21.5, 196.5)	.59
**AST (U/L)**	27.0 (21.7, 37.4)	33.1 (21.6, 117.5)	.20
**HBsAg (Log_{10} IU/ml)**	3.5 (2.9, 4.1)	3.3 (3.0, 3.8)	.61
**HBeAg positive status (%)**	101 (34.8)	5 (38.5)	.79
**HBV DNA (Log_{10} IU/ml)**	3.7 (2.7, 6.7)	3.7 (2.8, 6.4)	.89
**Fibrosis stage**			.03
**S0**	56 (19.3)	2 (15.4)	
**S1**	141 (48.6)	3 (23.1)	
**S2**	65 (22.4)	5 (38.5)	
**S3**	19 (6.6)	1 (7.7)	
**S4**	9 (3.1)	2 (15.4)	
**Severe fibrosis (S ≥2, %)**	93 (32.1)	8 (61.5)	.03
**Advanced fibrosis (S ≥3, %)**	28 (9.7)	3 (23.1)	.12
**Cirrhosis (S4, %)**	9 (3.1)	2 (15.4)	.02

ALT, alanine aminotransferase; anti-HBs, hepatitis B core antibody; AST, aspartate aminotransferase; HBeAg, hepatitis B e antibody; HBsAg, hepatitis B surface antigen; HBV, Hepatitis B virus; PLT, platelet.

© 2022 Wang J et al. *JAMA Network Open*.

eFigure. Flowchart of Patient Selection in Each Cohort

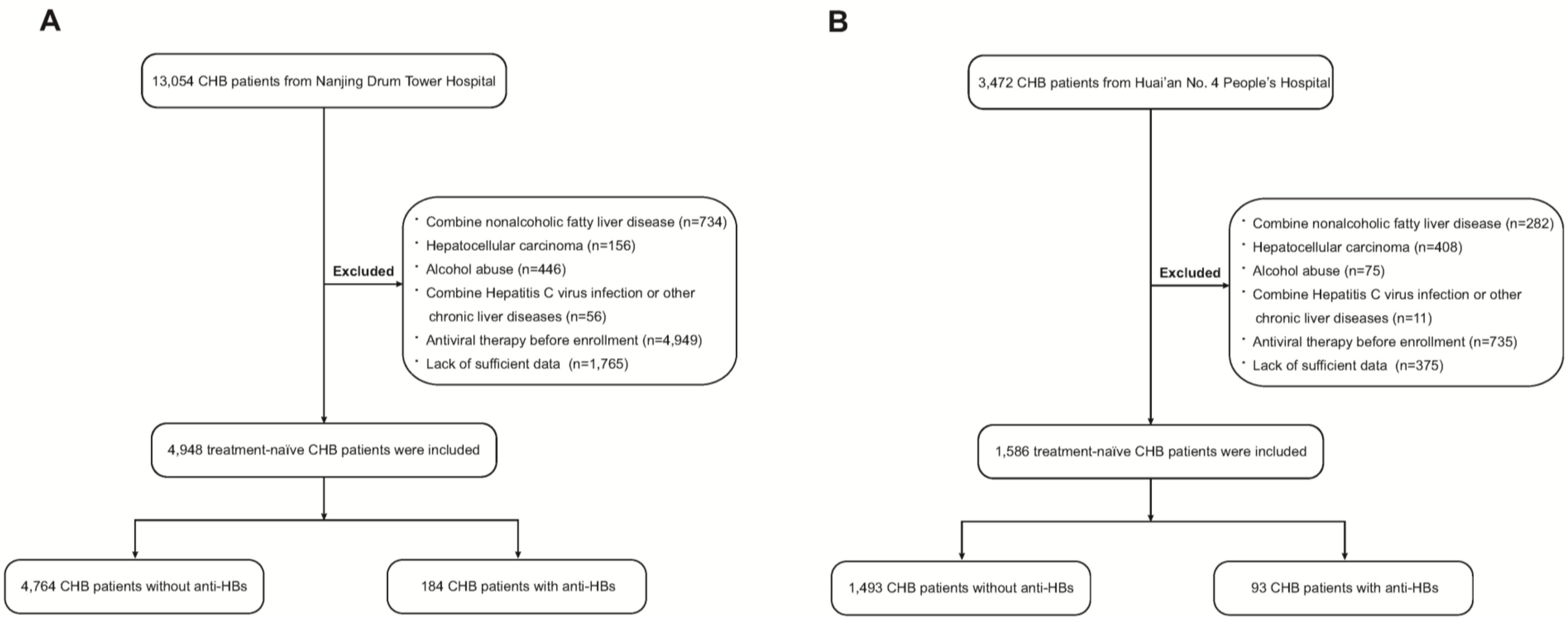

© 2022 Wang J et al. *JAMA Network Open*.
